# Overexpression of Lamin B Receptor Results in Impaired Skin Differentiation

**DOI:** 10.1371/journal.pone.0128917

**Published:** 2015-06-08

**Authors:** Agustín Sola Carvajal, Tomás McKenna, Emelie Wallén Arzt, Maria Eriksson

**Affiliations:** Department of Biosciences and Nutrition, Center for Innovative Medicine, Karolinska Institutet, Huddinge, SE-141 83, Sweden; University of Maryland School of Medicine, UNITED STATES

## Abstract

Hutchinson-Gilford progeria syndrome (HGPS) is a rare segmental progeroid disorder commonly caused by a point mutation in the *LMNA* gene that results in the increased activation of an intra-exonic splice site and the production of a truncated lamin A protein, named progerin. In our previous work, induced murine epidermal expression of this specific HGPS *LMNA* mutation showed impaired keratinocyte differentiation and upregulated lamin B receptor (LBR) expression in suprabasal keratinocytes. Here, we have developed a novel transgenic animal model with induced overexpression of LBR in the interfollicular epidermis. LBR overexpression resulted in epidermal hypoplasia, along with the downregulation and mislocalization of keratin 10, suggesting impaired keratinocyte differentiation. Increased LBR expression in basal and suprabasal cells did not coincide with increased proliferation. Similar to our previous report of HGPS mice, analyses of γH2AX, a marker of DNA double-strand breaks, revealed an increased number of keratinocytes with multiple foci in LBR-overexpressing mice compared with wild-type mice. In addition, suprabasal LBR-positive cells showed densely condensed and peripherally localized chromatin. Our results show a moderate skin differentiation phenotype, which indicates that upregulation of LBR is not the sole contributor to the HGPS phenotype.

## Introduction

Hutchinson-Gilford progeria syndrome (HGPS) is a very rare genetic disease with multiple clinical characteristics of premature aging (OMIM 176670). Children born with HPGS typically appear normal at birth, but within a year, they begin to display disease symptoms, including failure to thrive, severe growth retardation, and sclerodermatous skin changes. Effects of accelerated aging, including hair loss, diminished subcutaneous fat, cardiovascular disease, and skeletal abnormalities, become more prominent as the disease progress [1]. On average, death occurs at the age of 13 from heart attack or stroke [2]. HGPS is classified as a laminopathy [3] since it is caused by mutations in the *LMNA* gene, which encodes the lamin A and C proteins by alternative splicing. Lamins are the major components of the nuclear lamina, a meshwork of proteins underlying the inner nuclear membrane [3], and they can be classified based on different properties into A (lamin A and C) and B types (lamin B1 and B2) [4], which are encoded by separate genes: *LMNB1* and *LMNB2* [5]. Lamins not only provide structural support to the nucleus [6], but they also have important roles in essential cellular processes such as nucleo-cytoskeletal coupling, chromatin organization, epigenetic modifications, DNA replication, transcriptional regulation and repair, and the response to oxidative stress [3,7,8]. The expression pattern of lamin A and C is specific to each tissue and effects differentiation [5, 9] whereas B-type lamins are expressed in all cell types and in all stages of development. Apart from lamins, other transmembrane proteins, such as lamin B receptor (LBR), lamin-associated protein 2β (LAP2β) and emerin (EMD), are also part of the nuclear lamina [5].

The majority of HGPS cases are associated with a *de novo* dominant point mutation in exon 11 of the *LMNA* gene (c.18245C>T, p.G608G) [10, 11]. This HGPS mutation does not cause an amino acid change (G608G), but partially activates a cryptic splice donor site and leads to the in-frame deletion of 150 nucleotides within the prelamin A mRNA. This truncated prelamin A mRNA is then translated into a protein with an internal deletion of 50 amino acids, named progerin (or lamin AΔ50) [10]. This protein remains only partially processed and is C-terminally farnesylated, a normal lamin A processing step that is usually transient [12]. As a consequence, progerin disrupts the structure and properties of the lamina, thereby causing HGPS. Progerin can also be found in normal aging, at both the RNA and protein levels, and there is evidence that progerin levels may increase with age and be responsible for some of the cellular defects associated with aging, which are reminiscent of those observed in HGPS patients [13–17]. The cellular defects that have been associated with HGPS include nuclear blebbing, thickening of the nuclear lamina, clustering of nuclear pores and, interestingly, loss of peripheral heterochromatin [13].

LBR is an integral nuclear membrane protein whose N- and C-termini are located in the nucleoplasm and inner nuclear membrane, respectively [18]. Recent studies have implicated LBR and lamin A/C in the peripheral localization of the chromatin. LBR mediates the peripheral localization of chromatin during early development or in less differentiated cells, while lamin A/C mediates the peripheral localization of chromatin in differentiated cells [19]. This same group also showed that ectopic LBR expression could compensate for the loss of lamin A/C in differentiated cells [19]. In a recent study from our laboratory, in which the transgenic expression of the HGPS mutation, *LMNA* c.1824C>T, was induced in the skin during embryogenesis and early postnatal development [20], we analyzed the effects of expressing the HGPS mutation during the formation of the epidermal barrier and early postnatal skin development. Expressing the HGPS mutation in basal cells of the interfollicular epidermis did not have a significant effect on the formation of the epidermal barrier; however, as progerin accumulated, and with the birth of the mice, progressive skin disease resulted in halted skin development as early as postnatal day four and premature death by postnatal week two [20]. Postnatal skin sections taken from progeroid animals also revealed a significant fraction of cells with upregulated LBR expression. This upregulated LBR expression coincided with aberrant DNA distribution, in agreement with DNA condensation, in the suprabasal cells of the progeroid mice. These characteristics were not evident in suprabasal cells from wild-type littermate controls [20].

To further examine the contribution of LBR to keratinocyte differentiation, as well as the progeroid skin phenotype observed in our previous study, we have induced the expression of the LBR gene in the basal cells of the interfollicular epidermis using a combination of previously published transgenic mice models [21,22]. Using this novel transgenic mouse model, we explored the hypothesis that LBR overexpression in epidermal cells might lead to a progeroid skin phenotype and analyzed the effects of LBR overexpression on nuclear structure and chromatin organization.

## Material and Methods

### Ethics Statement

This study was performed in accordance with the guidelines for working with experimental animals set by the Karolinska Institute and all efforts were made to minimize animal suffering. All animal studies were approved by the Stockholm South Ethical review board (Dnr. S101–12 and S10-14 to Maria Eriksson).

### Transgenic mice

Transgenic mice were housed under a 12-h light-dark cycle, 20–22°C temperature and 50–60% humidity in a pathogen-free animal facility within Karolinska University Hospital, Huddinge, Sweden. The animals were supplied with RM3 pellets (Scanbur) and water *ad libitum*. Heterozygous Tet-O-lbr mice [21] were intercrossed with heterozygous K5tTA mice [22], and offspring were genotyped in accordance with previously described procedures [23,21]. Throughout the paper, K5+/LBR+ refers to Tet-O-lbr and K5tTA bitransgenic mice. As control mice, we used littermates that did not have either transgene, K5-/LBR-, which were referred to as wild-type mice. Body weights were measured weekly from postnatal week 3 to 5 (*n* = 39).

### Tissue collection and processing

Animals were sacrificed by an overdose of isoflurane. Paws and dorsal skin were collected and fixed in 4% paraformaldehyde (pH 7.4) at +4°C overnight. Samples were dehydrated and embedded in paraffin. Four-micrometer thick sections were stained with hematoxylin and eosin.

### Immunofluorescence and imaging

Four-micrometer thick sections were rehydrated, followed by antigen retrieval and blocking. Sections were incubated overnight at 4°C in the following primary antibodies: guinea pig serum against LBR at a dilution of 1:500 [24], anti-lamin A/C (1:75, N18, Santa Cruz) and anti-p16 (1:100, Santa Cruz). Antibodies for skin differentiation markers were as previously described [20]. Secondary antibody incubations were with Alexa 488-conjugated donkey anti-goat (1:500, A-11055, Life Technologies), Alexa 546-conjugated goat anti-guinea pig (1:500, A-11074, Life Technologies), and Alexa 555-conjugated goat anti-mouse (1:500, A-2122, Life Technologies). Normal goat, donkey or rabbit serum, BSA or mouse-to-mouse blocking reagent (Scytek, Logan, UT, USA) was used for blocking. DAPI (Vector laboratories) or DRAQ5 (1:1000, ab109202, Abcam) were used for nuclear staining. Sections were mounted using ProLong Gold (Life Technologies). Imaging was performed using Nikon A1R and A1+ imaging systems, (Nikon corporation, Japan), and images were analyzed with NIS elements (Nikon Corporation, Japan). Epidermal thickness and the frequency of Ki67-positive cells were calculated using NIS elements software with wild-type and bitransgenic mice (*n* = 10). Both cells expressing LBR (defined as LBR high) and cells not expressing LBR (defined as LBR low) from the suprabasal cell layer were selected for DNA distribution analysis. An line intensity profile tool (NIS elements, Nikon Corporation, Japan) analysis was performed across the nuclei on DAPI-stained cells to analyze the DNA distribution relative to LBR expression. Peripheral DNA distribution was defined as cells that lacked any detectable DAPI staining within the majority of the center of the nuclear interior. The frequency of the DNA distribution in LBR high and low cells was calculated with at least 100 cells counted per group (*n* = 3 per sample group).

### Keratinocyte isolation and staining

Primary keratinocytes were isolated and processed for immunofluorescence as previously described [25]. Primary antibodies were guinea pig serum against LBR (1:500) [24] and anti-phospho-Histone H2A.X (γH2AX; 1:100, JBW301, Millipore) at 4°C overnight. Samples were then incubated with Alexa 546-conjugated goat anti-guinea pig (1:500, A-11074, Life Technologies) and Alexa 647-conjugated goat anti-mouse (1:500, A21236, Life Technologies) secondary antibodies and DAPI (1:1000 Vector laboratories). The sections were mounted using ProLong Gold (Life Technologies) and imaged on Nikon A1R and A1+ imaging systems (Nikon corporation, Japan). The number of cells with γH2AX foci was calculated from keratinocytes isolated from wild-type and bitransgenic mice (*n* = 6), with a minimum of 200 keratinocytes counted per animal.

### Western blot

Proteins were extracted from mouse skin, and Western blotting and protein quantification were performed as previously described [20]. Antibodies against LBR (1:2000), lamin A/C (1:75, N18, Santa Cruz Biotechnologies) or keratin 10 (1:5000, PRB-159P, Convance) and the corresponding secondary antibodies HRP conjugated rabbit anti-guinea pig (1:10000, Life Technologies), rabbit anti-goat (1:40000, Jackson Immuno Research) and goat anti-rabbit (1:50000, Jackson Immuno Research) were used. Relative protein levels were analyzed in extracts from skin samples from 8-week-old mice (*n* = 10).

### Quantitative RT-PCR

RNA was isolated from keratinocytes of 13-week-old wild-type and bitransgenic mice (*n* = 3 per genotype) using TriZol Reagent (Invitrogen, Carlsbad, CA, USA). Random hexamers and SuperScript II Reverse Transcriptase (Invitrogen, Carlsbad, CA, USA) were used to synthesize cDNA from 0.8 μg of total RNA. Primer sequences and conditions are available upon request. Relative qPCR was performed in accordance with previously described procedure [26, 25].

### Statistical analysis

Statistical analyses were performed using unpaired Student’s T-tests with a two-tailed 95% confidence interval. The values represent the mean ± SEM; *p*-values of 0.05 to 0.01 were considered significant (*), a *p*-value of 0.01–0.001 was indicated as ** and a *p*-value smaller than 0.001 was indicated as ***.

## Results

### Overexpression of lamin B receptor in the epidermis

**Fig 1:**
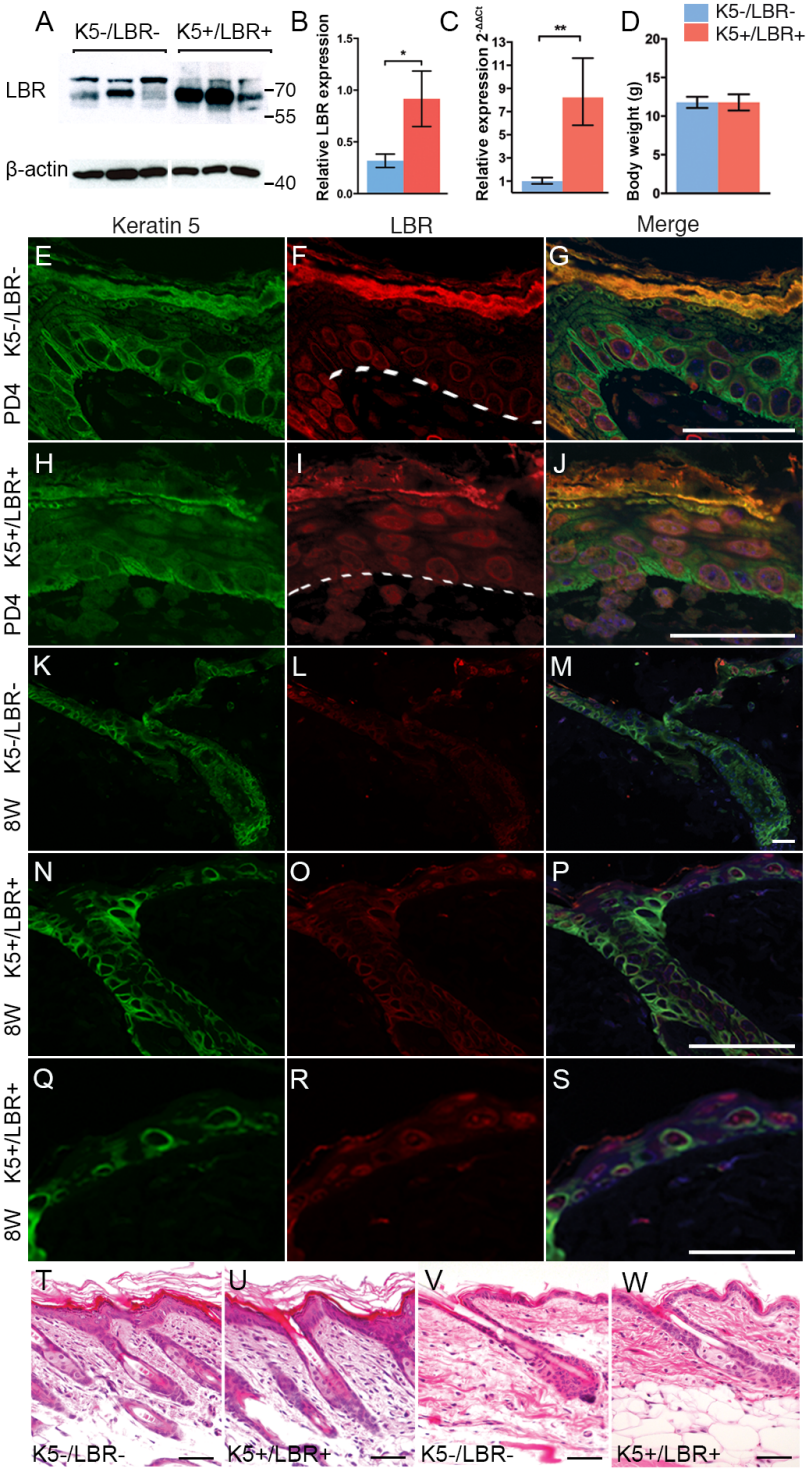
Epidermal LBR overexpression. A. A representative Western blot including 8-week-old K5-/LBR- (*n* = 3) and K5+/LBR+ (*n* = 3) protein extracts from dorsal skin. LBR migrates in two fragments of different molecular weight, as previously described, which may be explained by post transcriptional modifications in the processing of LBR [24]. B. The LBR fragments were quantified in dorsal skin of 8-week-old mice. Numbers and lines indicate molecular weight markers (kDa). C. Overexpressed LBR by relative qPCR in keratinocytes from 13-week-old K5-/LBR- (blue bars) and K5+/LBR+ mice (red bars). D. Body weights from week-5 K5-/LBR- (blue bars, *n* = 10) and K5+/LBR+ mice (red bars, *n* = 10). E-J. Keratin 5 (green) and LBR (red) in dorsal skin at postnatal day 4 (PD4, E-J) and week 8 (K-S). Overexpressed LBR in the basal and suprabasal layers (I-J, O-P, and R-S). T-W. Hematoxylin & eosin-stained dorsal skin from PD4 (T and U) and week 8 (V and W). Error bars indicate SEM (* *p*<0.05, ** *p*<0.01). Scale bars indicate 40 μm.

To explore the possible effects of LBR overexpression in skin and test the hypothesis that the upregulated LBR expression observed in the suprabasal cells of progeroid mice might interfere with the terminal differentiation of keratinocytes [20], we generated a mouse model by intercrossing Tet-O-lbr transgenic mice [21] with K5tTA mice [22] and analyzed the bitransgenic offspring for overexpression of LBR in the basal epidermal keratinocytes. Mice were born at the expected Mendelian frequencies, and there were no signs of embryonic lethality associated with LBR overexpression during embryogenesis (data not shown). Bitransgenic (K5+/LBR+) and wild-type (K5-/LBR-) mice were identified using standard genotyping protocols [22, 21]. The level of transgenic overexpression was quantified by performing Western blots on protein extracts from the dorsal skin of 8-week-old bitransgenic and wild-type mice using an anti-LBR antibody (Fig 1A). The average relative expression of LBR was 0.91 and 0.31 in bitransgenic and wild-type skin, respectively (Fig 1B), indicating that the average transgenic overexpression of LBR was 2.9 times higher in skin from K5+/LBR+ mice compared with K5-/LBR- mice (Fig 1B). Transgenic overexpression was analyzed at the RNA level by performing quantitative RT-PCR (q-PCR), which showed an 8-fold increase in the expression of LBR transcripts in K5+/LBR+ mice versus K5-/LBR- mice (Fig 1C). The difference in overexpression values observed between protein and RNA was likely dependent on differences in tissue heterogeneity for cells expressing the LBR transgene, given that the protein extracts were taken from dorsal skin, whereas the RNA was extracted from keratinocytes. Body-weight and morbidity analyses did not show any differences between K5+/LBR+ and K5-/LBR- mice (Fig 1D).

Immunofluorescence using an LBR antibody in dorsal skin from K5-/LBR- and K5+/LBR+ mice at postnatal day 4 and at postnatal week 8 confirmed the expression of LBR in the basal layer of the interfollicular epidermis and the hair follicles of the skin (Fig 1E–1S). Enhanced LBR expression was observed in keratinocytes of the basal and suprabasal layer at both time points in K5+/LBR+ mice (Fig 1I, 1O and 1R). Analyses of skin pathology in the dorsal skin from K5+/LBR+ mice at the two different postnatal time points (postnatal day 4 and postnatal week 8) did not show a significant difference compared with K5-/LBR- mice (compare Fig 1T and 1U with Fig 1V and 1W). No significant evidence for the characteristic intermediate skin phenotype seen in HGPS mice, including epidermal hyperplasia, marked hyperkeratosis, inflammatory cell infiltration, and moderate fibrosis of the dermis [23], was found.

### Epidermal hypoplasia in the paws of K5+/LBR+ mice

**Fig 2:**
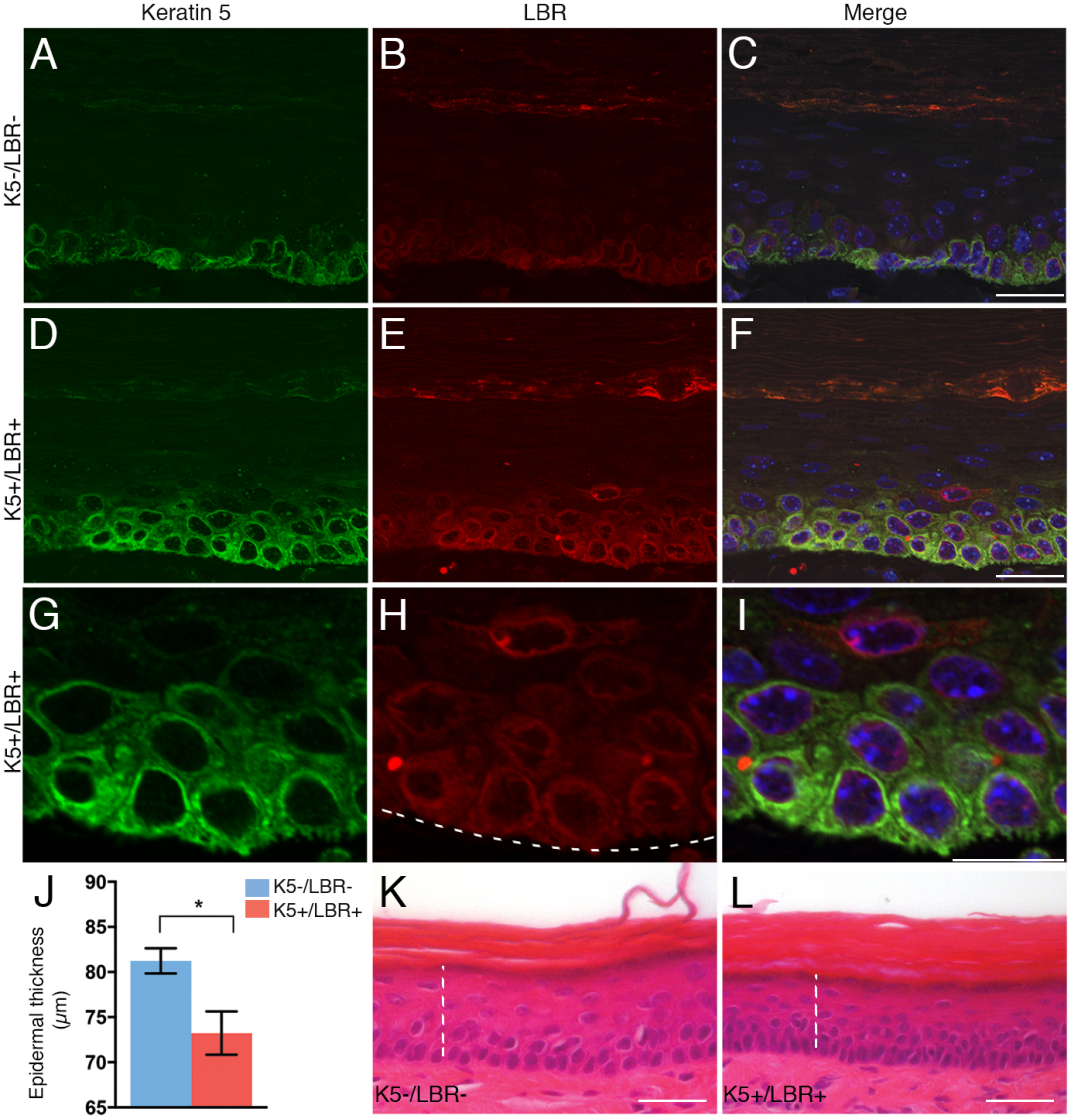
Overexpression of LBR results in hypoplasia of paw epidermis. A-I. Immunofluorescence of plantar paw with LBR (red), keratin 5 (green), and DNA (blue) in 8-week-old K5-/LBR- (A-C) and K5+/LBR+ mice (D-F). Normal expression of LBR in the basal cell layer and a few suprabasal cells (B). LBR overexpression and upregulated keratin 5 expression in basal and suprabasal keratinocytes (G-I). J-L. Measurement of the thickness of the paw epidermis in week-8 K5-/LBR- (blue) and K5+/LBR+ (red) mice. Dashed lines indicate region for measurement of epidermal thickness (basal cell layer to stratum granulosum) in K5-/LBR- (K) and K5+/LBR+ (L) mice, with at least 5 measurements per sample. Error bars indicate SEM (* *p*<0.05). Scale bars indicate 20 μm (C, F) and 10 μm (I).

LBR overexpression was evident via LBR-antibody immunofluorescence in both the thin and thick layers of the paw epidermis in K5+/LBR+ mice compared with K5-/LBR- mice (Fig 2A–2I, and data not shown). LBR expression was also observed in both the basal and suprabasal cells of the paw epidermis (Fig 2A–2I). Moreover, the layers expressing keratin 5, a basal cell marker, were expanded in the K5+/LBR+ mice compared with K5-/LBR- mice (*n* = 5 per group) (compare Fig 2A and 2D). Further histological analyses of skin pathology in the paw epidermis of 8-week-old mice showed a decreased thickness of the paw epidermis in K5+/LBR+ mice (basal to granular cell layers) compared with wild-type mice (Fig 2J–2L).

### Upregulation of S100A9 in keratinocytes from K5+/LBR+ mice

**Fig 3:**
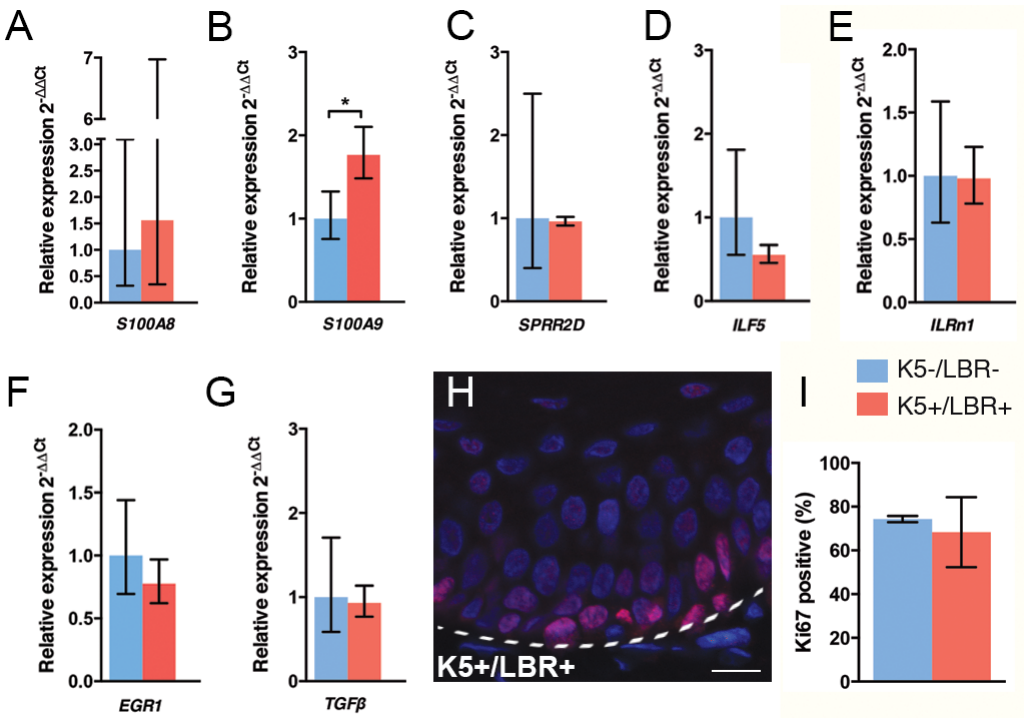
Increased S100A9 expression in K5+/LBR+ keratinocytes. A-G. Relative qPCR analysis of keratinocytes from week-13 K5-/LBR- (blue) and K5+/LBR+ (red) mice (*n* = 3 per group). (H) Analysis of keratinocyte proliferation, using Ki67 (red), and DNA-stained nuclei (blue) in paw epidermis of week-8 K5-/LBR- (blue) and K5+/LBR+ (red), (*n* = 3 per group). (I) No significant differences were observed between K5-/LBR- (blue) and K5+/LBR+ mice (red), (*n* = 3 per group). Error bars indicate SEM (* *p*<0.05). Scale bar indicates 20 μm.

Previous studies have shown the upregulation of inflammatory genes in skin from progeroid mice [25, 20]. NF-κB has been reported to be activated in DNA damaged and senescent cells, as well as being implicated in inflammation, aging and HGPS [27–29]. TGF-β is related to keratinocyte growth regulation and differentiation [30] and has been described to induce alopecia, epidermal hyperproliferation, dermal fibrosis, and inflammation in mice [31]. To further characterize the K5+/LBR+ mice for similarities to previously reported changes in the skin of progeroid mice, we examined the expression of both the NF-κB and TGF-β pathways by means of relative qPCR. Keratinocytes were extracted from the dorsal skin of 13-week-old K5+/LBR+ and K5-/LBR- mice. A significant upregulation of the inflammatory marker S100A9 (*p* = 0.033), was observed in K5+/LBR+ mice compared with wild-type mice (Fig 3B). There were no significant changes between K5+/LBR+ and K5-/LBR- mice for any of the other transcripts being assessed (Fig 3B–3G).

### LBR overexpression effects epidermal differentiation

**Fig 4:**
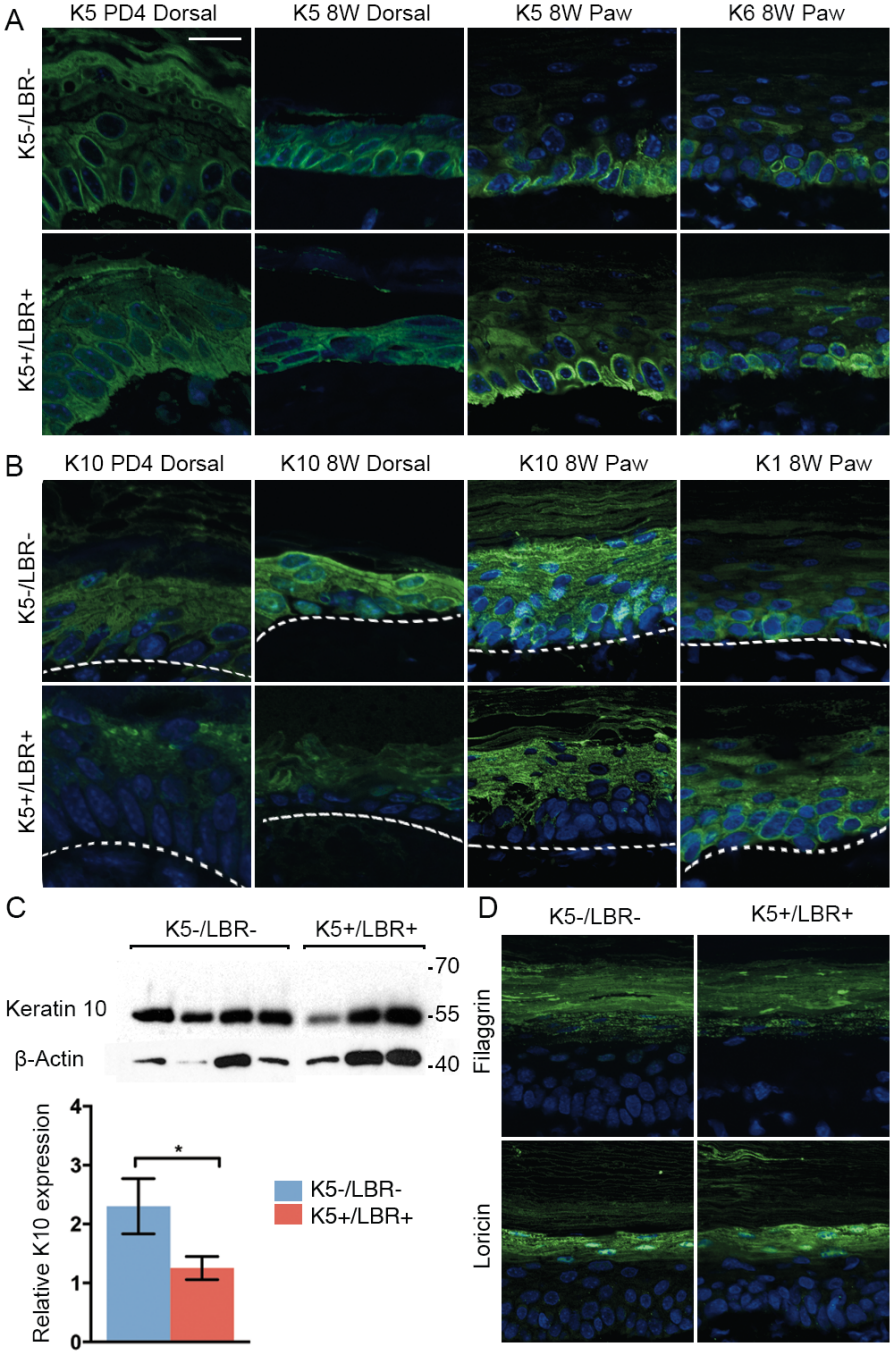
LBR overexpression results in keratin 5 upregulation and keratin 10 downregulation. A-B. Markers for epidermal differentiation (green) and DNA (blue), in dorsal skin from PD4 and week 8, and paw skin from week 8. Keratin 5 (K5) showed positive cells in the basal layer of the interfollicular epidermis; however, in K5+/LBR+ mice, K5 was also upregulated in cells of the suprabasal layer (A). Keratin 1 (K1) was found in the suprabasal layer, mainly in the spinous layer, while keratin 10 (K10) was located mainly in the suprabasal layer. In the K5-/LBR- mice, some cells of the basal layer showed K10, which was not evident in the K5+/LBR+ mice (B). Keratin 6 (K6). The dashed line marks the basal cell layer. C. A representative Western blot including 8-week-old K5-/LBR- (*n* = 4) and K5+/LBR+ (*n* = 3) protein extracts from dorsal skin. Keratin 10 protein expression was quantified by Western blot densitometry in K5-/LBR- (blue bars) and K5+/LBR+ (red bars) mice. Numbers and lines indicate molecular weight markers (kDa). D. Loricin and Filagrin (green) expression in the granular layer of paw skin from 8 weeks old mice. Error bars indicate SEM (* *p*<0.05). Scale bars indicate 20 μm.

To test if the proliferative turnover of keratinocytes in the differentiated regions of the paw epidermis was linked to LBR expression, we performed immunofluorescence for Ki67. The total number of Ki67-positive cells was not significantly different between the K5+/LBR+ and wild-type mice (Fig 3H and 3I), indicating that proliferative turnover might be more related to mechanical stress than to the overexpression of LBR. The gradual differentiation of keratinocytes from the basal layer to the skin surface creates the different layers of the epidermis [32]. The differentiated epidermis of the skin is a multi-layered stratified squamous epithelium, which maintains homeostasis via the proliferation of keratinocytes in the basal layer. To examine whether the expression of LBR during skin development alters epidermal differentiation, we performed immunofluorescence for differentiation markers in the dorsal skin at postnatal day 4 and postnatal week 8, and in the paw skin of 8-week-old mice (Fig 4). Keratin 5 was mainly localized in the basal cell layer of the epidermis in K5-/LBR- and K5+/LBR+ mice (Fig 4A). As mentioned previously, there was also a significant fraction of suprabasal cells showing keratin 5 expression in both the epidermis of the dorsal skin at PD4 and the paw epidermis in the K5+/LBR+ mice (Fig 4A). This effect could not be confirmed in 8-week old dorsal skin, since the mature epidermis is too thin to differentiate between layers. Analysis of keratin 6 in the paw epidermis did not reveal a significant difference between K5-/LBR- and K5+/LBR+ mice (Fig 4A).

Keratin 1 and 10 were localized in the suprabasal cell layers, mostly in the spinous layer of the epidermis, in both K5-/LBR- and K5+/LBR+ mice (Fig 4B). A few basal cells also showed keratin 1 and 10 expression in the K5-/LBR- mice; a similar pattern was also observed with keratin 1 in the basal cells of K5+/LBR+ mice (Fig 4B). However, keratin 10 expression was significantly decreased in the K5+/LBR+ mice compared with K5-/LBR- mice, and none of the basal cells showed expression of keratin 10 (Fig 4B). Significant K10 downregulation was observed when levels of this protein were quantified on dorsal skin from 8-week old wild-type and bitransgenic mice (*p* = 0.0157. Fig 4C). There was no significant difference in loricrin and filaggrin (markers for the granular layer) staining between the K5+/LBR+ and K5-/LBR- mice (Fig 4D).

### LBR overexpression does not effect the expression pattern of lamin A/C

**Fig 5:**
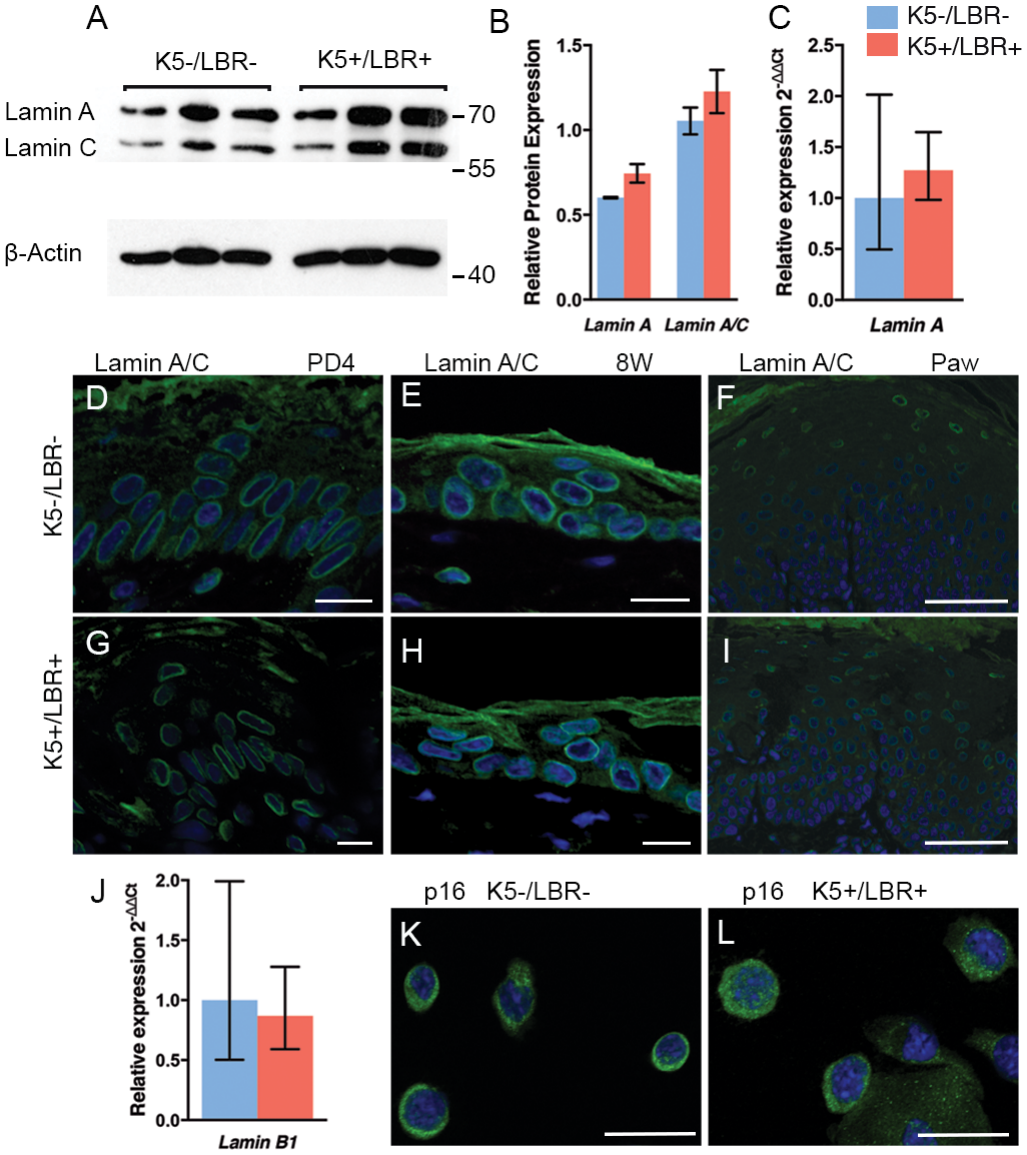
No changes in lamin A/C expression or signs of senescence upon LBR overexpression. A. A representative Western blot including 8-week-old K5-/LBR- (*n* = 3) and K5+/LBR+ (*n* = 3) protein extracts from dorsal skin. B. Quantification of lamin A/C from Western blots of protein extracts from dorsal skin of week-8 K5-/LBR- (blue) and K5+/LBR+ (red) mice. Numbers and lines indicate molecular weight markers (kDa). C. Relative qPCR of lamin A in primary keratinocytes in week-13 dorsal skin from K5-/LBR- (blue) and K5+/LBR+ (red) mice. D-I. Lamin A/C immunofluorescence (green) was positive in the basal layer and the terminally differentiated cells from the suprabasal layer. J. Lamin B1 expression by qPCR in primary keratinocytes from week-13 K5-/LBR- (blue) and K5+/LBR+ (red) mice. K-L. P16 (green) was assessed by immunofluorescence in keratinocytes extracted from week-13 dorsal skin. DAPI (blue). Scale bars indicate 20 μm (D-E, G-H) and 50 μm (F, I, K-L).

Solovei et al. have shown that LBR and lamin A/C expression occur sequentially and are temporally coordinated during development and cellular differentiation [19]. In the epidermis, basal cells detach from the basement membrane and gradually move upwards to the skin surface and undergo terminal differentiation. During this differentiation process, the cells go through a genomic reprograming that, among other things, includes the downregulation of *LBR* (expressed in basal layer cells) and the upregulation of lamin A/C as the cells reach their final differentiated state [19, 33]. In this study, we examined whether the overexpression of LBR could affect the expression levels of lamin A/C. Western blots using an antibody against mouse lamin A/C showed no changes in the relative expression of lamin A or lamin A/C in K5+/LBR+ mice compared with K5-/LBR- mice (Fig 5A and 5B). In addition, no significant difference was observed at the RNA level when lamin A was assessed by relative q-PCR in keratinocytes from dorsal skin at postnatal week 13 (Fig 5C). Lamin A/C was also assessed in differentiated skin using immunofluorescence in dorsal skin at postnatal day 4 and postnatal week 8, as well as the paw skin of 8-week-old mice, but no significant difference was observed between K5-/LBR- and K5+/LBR+ mice (Fig 5d–5I).

### No indication of premature senescence in K5+/LBR+ transgenic mice

Several previous studies have provided evidence that the expression of lamin B1 is downregulated in senescent cells [34–36]. However, relative qPCR analysis of lamin B1 in keratinocytes from 13-week-old K5-/LBR- and K5+/LBR+ mice did not show a significant difference (Fig 5J). Recently, increased expression of the well-known senescence marker p16 [37] has been reported in the premature aging of skin [38]. To analyze any changes in the expression of p16 in the skin, we performed immunofluorescence analysis using a specific antibody against p16 in primary keratinocytes from 13-week-old K5-/LBR- and K5+/LBR+ mice. The results did not show significant differences (Fig 5K and 5L).

### Peripheral DNA organization in cells expressing LBR

**Fig 6:**
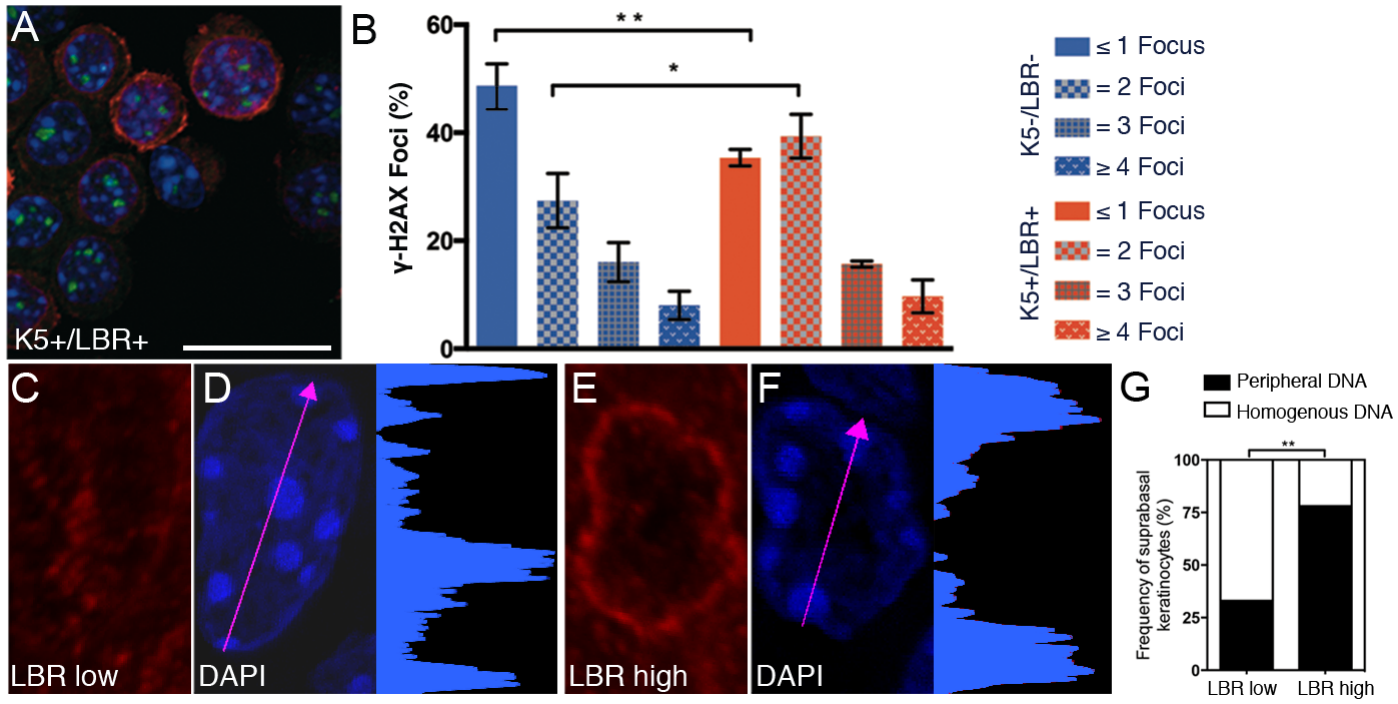
Increased DNA damage-related foci and peripheral DNA localization is associated with LBR overexpression. A-B. Primary keratinocytes from week-13 K5+/LBR+ (a) and K5-/LBR- mice were analyzed for the frequency of γH2AX foci (green), LBR (red) and DAPI (blue). C-F. The intensity profile of DAPI staining in suprabasal cells of the paw skin was examined in week-8 K5+/LBR+ mice. Cells that did not express LBR had a more homogenous intensity (C-D), whereas increased intensity of DAPI staining was seen at the nuclear periphery in cells that expressed LBR (E-F). D and F. The graph indicates fluorescence intensity of DAPI staining along the arrow. The histograms show the measured DAPI intensity. LBR (red). G. Quantification of the frequency of DNA distribution in LBR expressing (LBR high) and non-expressing (LBR low) cells of the suprabasal keratinocytes. Scale bar indicates 20 μm. *p<0.05, ** *p*<0.01.

Our previous studies of HGPS mice suggested increased DNA damage in keratinocytes [25]. Analysis of γH2AX, a marker for DNA double-strand breaks, showed an increased number of keratinocytes with 2 foci in LBR-overexpressing mice compared with wild-type mice (Fig 6A and 6B). This shift toward increased DNA double-strand breaks was also evident by the reduction of cells with 1 double strand break in K5+/LBR+ mice compared with K5-/LBR- mice (Fig 6B). In addition, LBR overexpression coincided with increased DNA staining intensity at the nuclear border (Fig 6C–6G), which is in agreement with our previous findings in HGPS mice [20]. While suprabasal cells that did not express LBR displayed more homogenous nuclear DNA staining (Fig 6C and 6D), the frequency of cells with peripheral DNA distribution in LBR expressing cells (LBR high) (Fig 6E and 6F) was significantly higher than those with peripheral DNA distribution in LBR non-expressing cells (LBR low) (*p* = 0.0018, Fig 6G).

## Discussion

In this study, we have analyzed the overexpression of LBR in basal and suprabasal cells and its possible contribution to a previously reported progeroid skin phenotype with impaired skin development. Previous results from our laboratory suggested that ectopic LBR expression in suprabasal cells of the epidermis may contribute to the impaired skin development phenotype in mouse expressing the *LMNA* c.1824C>T mutation (a mutation that has been associated with two different laminopathies, Restrictive Dermopathy and HGPS) during embryonic and early postnatal skin development [20]. Herein, we described our analyses of mice with 2.9-fold transgenic overexpression of LBR, which was observed in both basal and suprabasal keratinocytes of the epidermis. Analyses of skin pathology at multiple time points in the dorsal skin and paw epidermis did not reveal any significant changes, except for epidermal hypoplasia in the paw epidermis. This difference could not be explained by increased cell proliferation since there were no changes in Ki67 expression between the groups, but it might likely be caused by a different migration pattern of cells from the basal layer to the more external layers of the epidermis.

Staining for markers of skin differentiation revealed a multi-cell layer of keratin 5-positive cells in addition to mislocalized and downregulated expression of keratin 10, which are indicative of impaired differentiation. These results are in partial agreement with our previous study of HGPS mice, which showed suprabasal expression of keratin 5 in dorsal skin [25, 20]. However, the downregulation of keratin 10 has not previously been reported in HGPS mice [25,20]. Previous studies of keratin 10 knockout mice have shown a compensatory role for keratin 1 in addition to suprabasal keratin 5 and keratin 14 expression [39, 40].

In this study, we have shown that induced expression of LBR in the basal epidermis results in enhanced LBR expression and altered DNA distribution in suprabasal cells. We also noticed an increased number of cells with 2 foci of double-strand breaks in K5+/LBR+ compared with wild-type mice. This is in agreement with a recent study in which chromatin condensation was suggested to induce DNA damage responses [41]. Our results are in agreement with previous results suggesting that LBR mediates the peripheral localization of heterochromatin [19] because the overexpression of LBR in more differentiated suprabasal keratinocytes induced greater peripheral localization of the DNA.

Even though a mild increment in the DNA damage was seen in our model, no signs of premature senescence were found. Previous studies have shown that loss of lamin B1 is a marker for senescence [34–36]. Here we report no changes in lamin B1 expression, even though lamin B1 is a partner of LBR [42]. This is in agreement with the analysis of lamin B1 expresssion in the skin of our mice with expression of the *LMNA* c.1824C>T mutation, which did not show significant difference compared to wild-type mice [20]. However, analysis of skin sections from HGPS mice did emphasize a role for lamin B in the early stages of progeroid disease development [20]. While loss of B-type lamins does not affect skin development [43], complete loss of all nuclear lamins in the basal layer of the epidermis displayed ichthyosis, impaired development of skin and hair follicles and abnormal cell morphology [44].

While the HGPS mice developed a severe skin phenotype already within their first week of life [20], the less severe phenotype presented by the LBR mice reported herein would argue for a less significant role of LBR in the progeroid disease development. However, and as suggested by Solovei and colleagues [19], that the persisting suprabasal LBR expression that was seen in the epidermis of their *Lmna*-/- mice compensated for the loss of lamin A/C, our results support a similar role for the upregulated LBR expression to compensate for a non-functional lamina in the HGPS mice. Taken together, our results indicate that LBR is required in proper amounts in the basal layer of the epidermis to maintain normal skin differentiation, and that it has an important role in the regulation of keratin 10 expression.

## Supporting Information

S1 Table Arrive Checklist. (PDF)
